# Cellulosic ethanol production by natural bacterial consortia is enhanced by *Pseudoxanthomonas taiwanensis*

**DOI:** 10.1186/s13068-014-0186-7

**Published:** 2015-01-23

**Authors:** Ran Du, Jianbin Yan, Shizhong Li, Lei Zhang, Sandra Zhang, Jihong Li, Gang Zhao, Panlu Qi

**Affiliations:** Institute of New Nuclear and New Energy Technology, Tsinghua University, Beijing, 100084 China; Beijing Engineering Research Center for Biofuels, Tsinghua University, Beijing, 100084 China; The Tsinghua University-Peking University Center for Life Sciences, MOE Key Laboratory of Bioinformatics, School of Life Sciences, Tsinghua University, Beijing, 100084 China; Research Institute of Petroleum Processing, Beijing, 100000 China

**Keywords:** Natural consortium, Biomass, *Pseudoxanthomonas taiwanensi*s, Ethanol, Cellulose

## Abstract

**Background:**

Natural bacterial consortia are considered a promising solution for one-step production of ethanol from lignocellulose because of their adaptation to a wide range of natural lignocellulosic substrates and their capacity for efficient cellulose degradation. However, their low ethanol conversion efficiency has greatly limited the development and application of natural bacterial consortia.

**Results:**

In the present study, we analyzed 16 different natural bacterial consortia from a variety of habitats in China and found that the HP consortium exhibited relatively high ethanol production (2.06 g/L ethanol titer from 7 g/L α-cellulose at 55°C in 6 days). Further studies showed that *Pseudoxanthomonas taiwanensis* played an important role in the high ethanol productivity of HP and that this strain effectively boosted the ethanol production of various other natural bacterial consortia. Finally, we developed a new consortium, termed HPP, by optimizing the proportion of *P. taiwanensis* in the HP consortium to achieve the highest ethanol production reported for natural consortia. The ethanol conversion ratio reached 78%, with ethanol titers up to 2.5 g/L.

**Conclusions:**

In the present study, we found a natural bacterial consortium with outstanding ethanol production performance, and revealed an efficient method with potentially broad applicability for further improving the ethanol production of natural bacterial consortia.

**Electronic supplementary material:**

The online version of this article (doi:10.1186/s13068-014-0186-7) contains supplementary material, which is available to authorized users.

## Background

Lignocellulose is the most widespread and abundant source of carbon in nature, and it is considered the preferred biomass for the production of ethanol, as it has significant benefits for agriculture, the environment, renewable energy development, and national security [1,2]. However, the main technological impediment to more widespread utilization of lignocellulose for ethanol production has been the lack of low-cost technologies to overcome the recalcitrance of its chemical structure, which is composed of closely intertwined cellulose, hemicellulose, and lignin [2,3].

Direct conversion of lignocellulose to ethanol in a single processing step, known as consolidated bioprocessing (CBP), is a promising strategy for cost reduction because of its decreased operating requirements and the elimination of exogenous enzyme supplementation [4]. Substantial efforts have been undertaken to develop various approaches to improve the implementation of CBP. It has been reported that genetically engineered microbes, especially anaerobic strains, show efficient ethanol production and high product tolerance [5]. Moreover, an artificial consortium composed of genetically engineered strains could efficiently improve ethanol production capability [6]. However, engineered single strains and simple artificial consortia have thus far exhibited a limited substrate range, unstable fermentation performance, and high equipment and operational costs [3,7-9].

In contrast, natural bacterial consortia are innately capable of extensive conversion of lignocellulosic biomass [10]. Moreover, natural consortia offer other advantages, such as the ability to use a wide variety of natural lignocellulosic biomass substrates [11-13], outstanding self-stability, and few operational requirements such as pretreatment or sterilization [6,8,14-16]. However, it remains a challenge to decipher and optimize cellulosic ethanol production by natural bacteria consortia, as natural consortia are very complex and harbor multiple populations with overlapping niches formed by various uncultured and cultured bacteria with or without cellulolytic activities, thus generally resulting in poor ethanol production [3].

Here, we found that non-cellulolytic microbes play important roles in improving the cellulose fermentation performance of natural bacterial consortia, and create an efficient way to enhance ethanol production of natural bacterial consortia.

## Results

### Screening of natural bacterial consortia for cellulosic ethanol production

**Figure 1:**
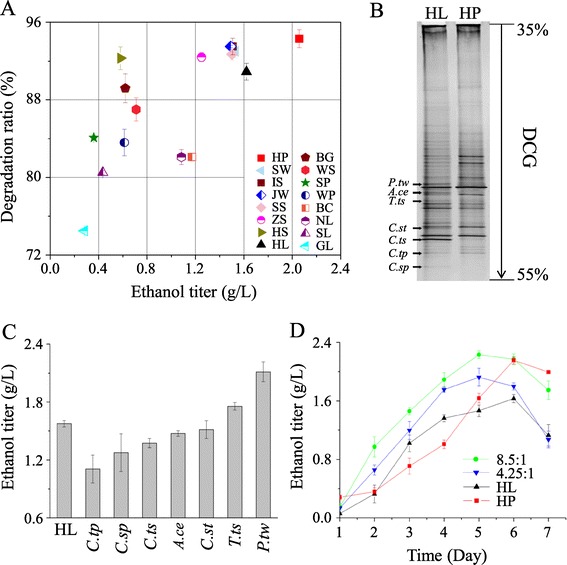
**Screening for natural bacterial consortia with high cellulosic ethanol production and identification of** ***P. taiwanensis*** **. (A)** Screening of consortia with cellulose degradation and ethanol production capabilities. In total, 16 consortia were collected from the locations listed in Additional file 1: Table S1, and their cellulose degradation and ethanol production performance was determined by culturing with 7 g/L α-cellulose for 6 days at 55°C. The error bars represent the SD (n = 3). **(B)** Community structure differences between HL and HP as determined using PCR-DGGE. Total DNA was extracted from fermentation cultures at the point when ethanol reached its highest titer, and partial 16S rDNA was then used for DGGE analysis. Left arrows indicate the strongest bands that were unique to the HP consortium; the corresponding strains are represented as follows: *C.tp* (*Clostridium thermopalmarium*), *C.sp* (*Clostridium sporogenes*), *C.ts* (*Clostridium thermosuccinogenes*), *A.ce* (*Acetivibrio cellulolyticus*), *C.st* (*Clostridium stercorarium*), *T.ts* (*Thermoanaerobacterium thermosaccharolyticum*), and *P.tw* (*Pseudoxanthomonas taiwanensis*). **(C)** The seven strains in B were cultured, and each was added to the HL consortium at a biomass ratio of 8.5:1 HL:strain. The co-fermentations were performed at 55°C for 7 days. The maximal ethanol titers are shown. The strain abbreviations under each column represent the co-fermentation of HL with the specified strains. HL represents fermentation by consortium HL without any added strains. The error bars represent the SD (n = 3). **(D)** *P. taiwanensis* boosts ethanol production by consortium HL in a dose-dependent manner. Co-fermentations were conducted with different proportions of *P. taiwanensis* and with α-cellulose as a carbon source at 55°C for 7 days with HL and HP as controls. The ratio in the legend represents the biomass proportion of the consortium and the single strains. The error bars represent the SD (n = 3).

To find natural consortia with efficient cellulosic ethanol production capabilities, we collected consortium samples from a wide variety of habitats in China (Additional file 1: Table S1) and isolated the consortia based on their cellulose degradation capacities at 55°C using α-cellulose as a carbon source. Consortia exhibiting α-cellulose degradation ratios of over 70% and stability over 10 generations of subcultivation were selected and subjected to ethanol fermentation with α-cellulose as the carbon source (Figure 1A). After fermentation for 6 days, the ethanol produced by the consortia was analyzed by high performance liquid chromatography (HPLC). Figure 1A shows that most of the consortia produced a low ethanol titer, ranging from 0.28 to 1.51 g/L and averaging 0.85 g/L. However, two consortia, HP and HL, showed significantly higher ethanol titers (2.06 g/L and 1.62 g/L, respectively). Moreover, in addition to α-cellulose, HP and HL also showed outstanding cellulosic ethanol production from sources of natural lignocellulose such as sweet sorghum stalks, indicating their potential for industrial cellulosic ethanol production using energy crops (Table 1).

**Table 1:** **Ethanol production of consortia with different carbon sources**

**Consortium**	α-Cellulose*	**Ethanol titer (g/L)**	Filter paper*	**Ethanol titer (g/L)**	Sweet sorghum stalks*	**Ethanol titer (g/L)**
HL	90.9 ± 1.49	1.62 ± 0.02	93.4 ± 1.11	1.70 ± 0.06	48.9 ± 2.31	0.85 ± 0.03
HLP	93.4 ± 2.09	2.23 ± 0.17	96.5 ± 0.67	2.32 ± 0.06	56.2 ± 2.49	1.01 ± 0.06
HP	94.3 ± 0.85	2.06 ± 0.05	98.1 ± 0.88	2.21 ± 0.05	69.3 ± 4.82	1.65 ± 0.09
HPP	95.2 ± 1.03	2.50 ± 0.08	98.4 ± 1.03	2.59 ± 0.08	74.7 ± 2.97	1.96 ± 0.05

*Degradation ratio of substrates.

We further analyzed the community structures of HP and HL at their highest ethanol titers with polymerase chain reaction and denaturing gradient gel electrophoresis (PCR-DGGE) assays. Surprisingly, most of the bands in the DGGE gel were identical between HP and HL, suggesting that the two consortia had similar microbial community compositions at their highest ethanol titer stages (Figure 1B). It was possible that slight differences in structure resulted in the different properties of HP and HL. Therefore, we further sequenced the dissimilar bands and identified seven cultivable microorganisms that existed only in the HP consortium (Table 2).

**Table 2:** **Sequence similarity analyses of DGGE bands 1 to 7 based on BLASTn comparison to the GenBank database**

**Number**	**BLAST result**	**Similarity (%)**
1	*Pseudoxanthomonas taiwanensis*	100
2	*Acetivibrio cellulolyticus*	95
3	*Thermoanaerobacterium thermosaccharolyticum*	99
4	*Clostridium stercorarium*	98
5	*Clostridium thermosuccinogenes*	99
6	*Clostridium thermopalmarium*	99
7	*Clostridium sporogenes*	100

To evaluate the roles of the HP-specific strains in the improved cellulosic ethanol production by HP, we individually co-fermented each of the strains with the HL consortium and measured the ethanol titer. Five of the seven single strains had negative effects on the fermentation performance of HL. However, the other two, particularly *P. taiwanensis* [17], exhibited a significant positive role in promoting ethanol production by the HL consortium (Figure 1C).

Furthermore, by adjusting the proportion of *P. taiwanensis* in the consortium, we were able to increase the maximum ethanol titer to 2.23 g/L (Figure 1D), which was 48.7% higher than the titer for the HL consortium alone and exceeded that for the HP consortium. These results suggested that *P. taiwanensis* was an important factor in the high ethanol production of the HP consortium.

### *P. taiwanensis* promotes cellulose utilization by consortia

**Figure 2:**
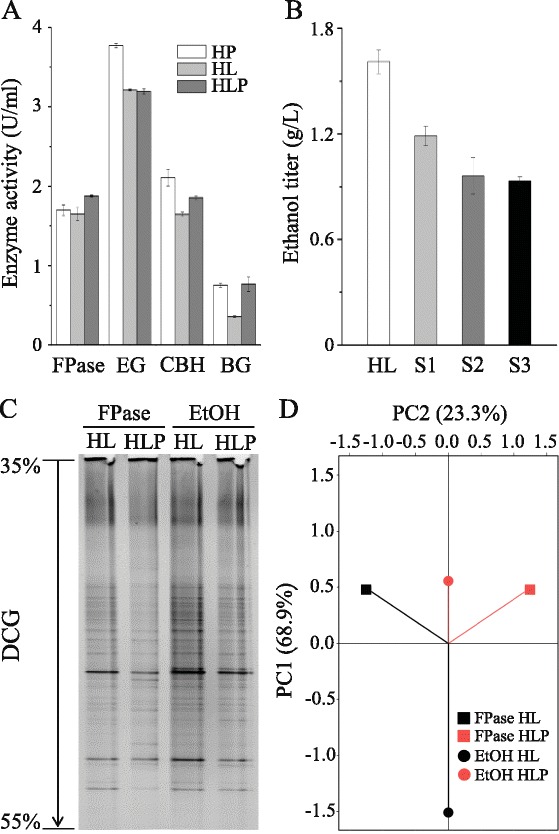
***P. taiwanensis*** **promotes cellulose utilization by consortia. (A)** Cellulase enzyme activities of HP, HL, and HLP. Fermentation was conducted with α-cellulose for 7 days at 55°C, and the cellulase enzyme activities of the consortia with and without co-cultured *P. taiwanensis* at an 8.5:1 biomass ratio were measured. The maximal activities are shown. FPase, filter paper activity; EG, endoglucanase; CBH, exoglucanase; BG, β-glucosidase. HLP represents the new consortium obtained by co-culturing HL and *P. taiwanensis*. The error bars represent the SD (n = 3). **(B)** Consortium performance cannot be improved by addition of β-glucosidase. β-glucosidase was added to consortium HL at final enzyme activities of 0.2 U/mL (S1), 0.8 U/mL (S2), and 1.6 U/mL (S3) at the beginning of fermentation. The control was fermentation by consortium HL without β-glucosidase addition (HL). The error bars represent the SD (n = 3). **(C)** PCR-DGGE analysis of HL and HLP at two stages, including the highest filter paper activity (represented as FPase) and highest ethanol titer (represented as EtOH). **(D)** Principal component analysis (PCA) of the PCR-DGGE data in C. PC1 and PC2 explained 68.9% and 23.3% of the total variance, respectively.

To determine the mechanism underlying the ethanol productivity improvement conferred on the consortium by *P. taiwanensis*, we tested whether the overall improvement resulted from ethanol production by *P. taiwanensis* itself. We found that *P. taiwanensis* could not produce ethanol under the same fermentation conditions with various monosaccharides and oligosaccharides involving glucose, xylose, sucrose, D-fructose, cellobiose, xylan, and cellulose, suggesting that *P. taiwanensis* lacks the capability for ethanol fermentation. Further analysis showed that *P. taiwanensis* had only β-glucosidase activity (0.48 U/ml) and no filter paper, endoglucanase, or exoglucanase activity, suggesting further that it would mainly play a role in the utilization of cellulose. Consistent with this finding, the HL consortium exhibited significantly lower β-glucosidase activity than the HP consortium, whereas the co-fermentation of HL with *P. taiwanensis* boosted its β-glucosidase activity by 2.13-fold, reaching a level similar to that of the HP consortium (Figure 2A).

Moreover, the increase in β-glucosidase activity conferred by *P. taiwanensis* could not be achieved by adding commercial β-glucosidase during fermentation. As shown in Figure 2B, the ethanol titer of the HL consortium was in fact reduced when β-glucosidase was added into the fermentation culture from the start. This result suggested that the contribution of *P. taiwanensis* to the consortium was complex, which was also consistent with the finding that *P. taiwanensis* increased the exoglucanase activity of the HL consortium while itself lacking exoglucanase activity. We therefore analyzed the changes in the consortium compositions resulting from the addition of *P. taiwanensis* by using PCR-DGGE (Figure 2C). Principal component analysis (PCA) based on the DGGE gel shown in Figure 2D revealed that the structures of the HL and HLP (co-fermentation of HL with *P. taiwanensis*) consortia were quite similar (the similarity reached approximately 80%) at the initial stage. However, at the highest ethanol titer, the similarity between HL and HLP declined to 57%, showing that the relative difference between the two community structures gradually increased as fermentation proceeded. These observations suggest that *P. taiwanensis* may have enhanced the growth of other cellulolytic bacteria in the HL consortium, thus indicating that *P. taiwanensis* serves a broader role in helping a natural consortium to utilize cellulose.

### *P. taiwanensis* strengthens ethanol production by various consortia

**Figure 3:**
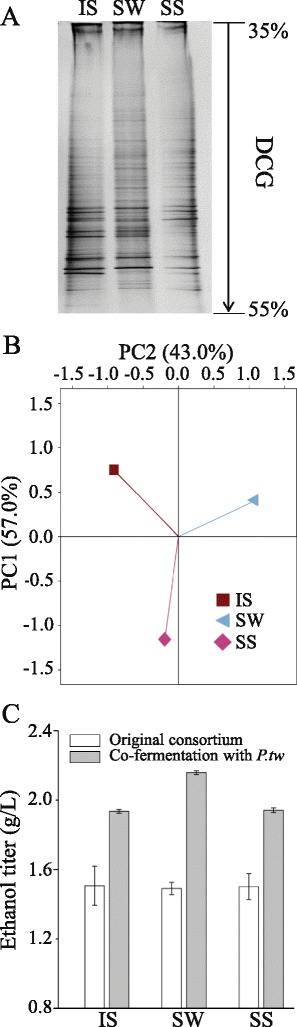
***P. taiwanensis*** **increases ethanol production by various consortia. (A)** IS, SW, and SS differed significantly in their community structure, as shown by PCR-DGGE analysis. **(B)** Principal component analysis (PCA) of the PCR-DGGE data in A. PC1 and PC2 explained 57.0% and 43.0% of the total variance, respectively. **(C)** Enhancing the ethanol production of consortia using *P. taiwanensis*. Fermentation was conducted at 55°C for 7 days with the original consortia as controls. Samples were collected each day to measure ethanol titers, and the highest titers are shown. The error bars represent the SD (n = 3).

To investigate whether co-fermentation with *P. taiwanensis* would boost the ethanol productivity of various other native consortia, we selected three other consortia from widely differing habitats, including IS isolate from steppe soils in Inner Mongolia, SW isolated from wheat straw in Shandong province, China, and SS isolated from sorghum stalks in Shandong province, China. The PCR-DGGE and PCA analyses shown in Figure 3A and B confirmed that the three consortia were significantly different in their bacterial community structures. These consortia were each co-fermented with *P. taiwanensis* at a biomass proportion of 8.5:1. After co-fermentation in anaerobic bottles at 55°C, the ethanol titers were evaluated; addition of *P. taiwanensis* promoted the ethanol productivity of all consortia. Compared to the controls, ethanol production was increased by 28.5% for IS, 44.8% for SW, and 29.3% for SS (Figure 3C). These results suggest that co-fermentation with *P. taiwanensis* is an effective method with potentially broad applicability for increasing the cellulosic ethanol production of native consortia.

**Figure 4:**
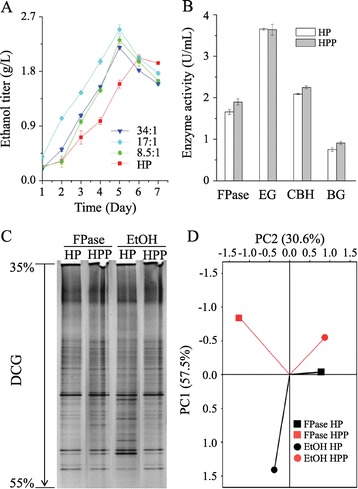
**The strengthened consortium HP with** ***P. taiwanensis*** **(HPP) exhibits increased ethanol production capability. (A)** *P. taiwanensis* boosts ethanol production by consortium HP in a dose-dependent manner. Co-fermentations were conducted with different proportions of *P. taiwanensis* and with α-cellulose as a carbon source at 55°C for 7 days, with HP as a control. The ratio in the legend represents the biomass ratio between the consortium and the single strain. The error bars represent SD (n = 3). **(B)** Cellulase enzyme activity of HP and HPP. Fermentation was conducted with α-cellulose at 55°C for 7 days, and the cellulase enzyme activities of the consortium with and without *P. taiwanensis* co-culture at a 17:1 biomass ratio were measured; the highest activities are shown. FPase, filter paper activity; EG, endoglucanase; CBH, exoglucanase; BG, β-glucosidase. HPP represents the new consortium obtained by co-culture of HP and *P. taiwanensis*. The error bars represent the SD (n = 3). **(C)** PCR-DGGE analysis of HP and HPP at two stages, including the highest filter paper activity (represented as FPase) and highest ethanol titer (represented as EtOH). **(D)** Principal component analysis (PCA) of the PCR-DGGE data in C. PC1 and PC2 explained 57.5% and 30.6% of the total variance, respectively.

Based on this idea, we further optimized the HP consortium and found that the highest ethanol titer, 2.5 g/L from α-cellulose, was achieved at a biomass ratio of 17:1 between the HP consortium and *P. taiwanensis* (Figure 4A). Compared with the original HP consortium, the optimized HP that contained *P. taiwanensis* (HPP consortium) showed a 21.5% increase in β-glucosidase activity and slight improvements in exoglucanase and filter paper activities (Figure 4B). A community structure analysis (Figure 4C and D) further revealed an 18% difference between HP and HPP at the point of the highest ethanol titer. Given that the HP consortium alone included a certain amount of *P. taiwanensis*, these results demonstrated that addition of *P. taiwanensis* at the correct biomass ratio could further improve the cellulose utilization of natural consortia that already possessed the strain. Moreover, the HPP consortium, with an optimized *P. taiwanensis* concentration, exhibited significant improvement in cellulose degradation and conversion of filter paper and sweet sorghum stalks (Table 1), suggesting that HPP has potential for industrial ethanol production utilizing natural lignocellulosic substrates.

## Discussion

In the present study, we found that *P. taiwanensis* enhanced cellulose utilization by various natural consortia (Figures 1, 2, 3, and 4). The main reason for the enhancement is most likely the production of β-glucosidase by *P. taiwanensis* (Figure 2). These results suggest that β-glucosidase is important and that its production is often a rate-limiting step in natural bacterial consortia.

Previous studies with single strains and purified enzymes have shown that β-glucosidase has essential roles in removing cellobiose during cellulose hydrolysis [1,18-20]. The observation that exoglucanase activity increased when *P. taiwanensis* was added to consortia (Figures 2A and 4B) suggests the possibility that the presence of *P. taiwanensis* resulted in the generation of β-glucosidase, thereby promoting cellobiose digestion and reducing metabolite repression of exoglucanase in consortia.

Simple supplementation of β-glucosidase in the fermentation process did not improve performance (Figure 2B). This finding indicates that β-glucosidase must be synergistically produced and is likely dynamically regulated with other glycoside hydrolase enzymes, including endoglucanases and exoglucanase, during ethanol production by the growth and fermentation of a bacterial consortium.

In addition, previous studies characterizing *P. taiwanensis* found that the surface charge of the bacteria could efficiently aggregate microorganisms with the raw materials for papermaking [21], suggesting that *P. taiwanensis* may change the fermentation microenvironment by affecting the contact between the cellulose substrates and bacteria in the consortium.

We also successfully developed a new consortium, termed HPP, by using HP as the base consortium and optimizing the concentration of *P. taiwanensis*. Compared with reported consortia displaying 30% to 99% filter paper degradation ratios and 0.02 g/L to 1.6 g/L ethanol titers [10-13,16,22], HPP exhibited high filter paper degradation (99%) and a high cellulosic ethanol production capacity (2.59 g/L), as shown in Table 3. Furthermore, fermentation of sweet sorghum vinasse performed with HPP yielded a high ethanol titer (Table 1), demonstrating its ability to convert natural lignocellulose [10-16,22].

**Table 3:** **Comparison of the filter paper degradation ratios (%) and ethanol titers of HPP and various other bacterial consortia**

**Consortium**	**Time (days)**	**T (°C)**	**Substrate concentration (g/L)**	**Filter paper degradation (%)**	**Ethanol titer (g/L)**	**References**
MC1	4	50	10	79	1.56	Haruta *et al.* (2002) [16]
EMSD13	4	50	NS	85	NS	Lv *et al.* (2008) [12]
MC3F	7	50	10	55	NS	Wongwilaiwarin *et al.* (2010) [10]
H-C	8	40	10	81	0.09	Feng *et al.* (2011) [11]
H-D	8	40	10	55	NS	Feng *et al.* (2011) [11]
H-J	8	40	10	40	NS	Feng *et al.* (2011) [11]
H-S	8	40	10	30	NS	Feng *et al*. (2011) [11]
WCS-6	3	50	5	99	NS	Wang *et al.* (2011) [22]
SQD-1.1	3	30	2	NS	NS	Gao *et al.* (2014) [35]
SV79	7	42.5	10	NS	0.12	Zhao *et al.* (2014) [13]
HPP	3	55	5	99	2.59	This study

NS: Not reported.

The final, high-ethanol-titer natural consortium HPP benefited from two factors. First, an appropriate proportion of *P. taiwanensis* was used for co-fermentation with the HP consortium. We found that the *P. taiwanensis*-mediated enhancement of ethanol titer occurred only within a proper range. Too little *P. taiwanensis* had no obvious effect, and too much had a negative effect. Second, we started with a well-balanced original consortium. We noticed that ethanol production by HPP was significantly higher than that by HLP. One explanation could be that the original HP consortium contained extra strains with polysaccharide hydrolytic enzymes, such as *Acetivibrio cellulolyticus* [23], *Thermoanaerobacterium thermosaccharolyticum* [18], and *Clostridium stercorarium* [24], which could result in higher lignocellulose utilization. With these two advantages, the ethanol yield of the HPP consortium reached 0.36 g/g carbon source and 0.28 g/g carbon source with α-cellulose and sweet sorghum vinasse, respectively. Future work will involve deciphering the HPP consortium and regulating its metabolic pathways for better ethanol production by the addition of new strains or through metabolic engineering.

## Conclusion

In the present study, we evaluated the direct conversion of cellulose to ethanol by 16 natural bacterial consortia collected from a variety of habitats in China. We found that the best consortium (consortium HP) produced a 2.06 g/L ethanol titer from 7 g/L α-cellulose or a 1.65 g/L ethanol titer from 7 g/L sweet sorghum stalks after 6 days at 55°C. By analyzing the structure of the consortia, we found that *P. taiwanensis* played an important role in the high ethanol productivity of consortium HP. Further experiments suggested that *P. taiwanensis* functions by producing β-glucosidase and by regulating other cellulolytic bacteria in the consortium. Addition of *P. taiwanensis* to several other natural consortia increased their ethanol titers, demonstrating that this was an efficient method with potentially broad applicability for promoting lignocellulosic ethanol production by natural bacterial consortia. Moreover, by optimizing the proportion of *P. taiwanensis* in consortium HP, we developed a new consortium, termed HPP, which produced a 2.5 g/L ethanol titer and 78% ethanol conversion ratio using 7 g/L α-cellulose. These are the highest values yet reported for ethanol production by a natural consortium, suggesting that symbiotic microbial communities might represent an economic and feasible technology for cellulosic ethanol production.

## Materials and methods

### Consortium screening

Soil and humus samples were collected from different areas in China with various types of climates and different lignocellulosic substrates, as shown in Additional file 1: Table S1. Samples (5 g) were added to 100 mL of autoclaved modified peptone cellulose solution (PCS) [25] medium (1 g of yeast extract, 5 g of peptone, 5 g of NaCl, 2.5 g of CaCO_3_, 0.5 mg of ZnSO_4_, 0.05 mg of MnSO_4_, 0.05 mg of CuSO_4_, 0.05 mg of CoSO_4_, 0.05 mg of Na_2_B_4_O_7_, and 0.05 mg of NaMoO_4_ per liter) in 250-mL flasks with 0.7 g of α-cellulose (α-cellulose C8002, Sigma; Sigma-Aldrich Corp., St. Louis, MO, USA) as a carbon source and were incubated under static conditions at 55°C. After subculturing by sequential transfer 10 times in PCS medium every 5 days, the consortia with cellulose degradation values above 70% were selected and used in subsequent experiments. Sweet sorghum vinasses were obtained from sweet sorghum stalks subjected to advanced solid-state fermentation and pretreated as described by Li *et al.* [26].

### Substrate degradation ratio measurement

The residual solid cellulosic substrates were washed with acetic-nitric reagent (1 M) and water as described in Haruta *et al.* [16], and the weight of the residual lignocellulose was then measured, using blank medium as a control. The degradation ratio of the substrates was defined as the ratio of the weight of degraded substrates compared to the weight of total substrates added at the beginning of fermentation (%) and calculated by the following formula:

$$ \mathrm{Degradation}\ \mathrm{ratio}\ \mathrm{of}\ \mathrm{substrates}=1\kern0.5em -\kern0.5em \frac{\mathrm{m}\left(\mathrm{Residual}\ \mathrm{lignocellulose}\right)}{\mathrm{m}\left(\mathrm{Total}\ \mathrm{lignocellulose}\ \mathrm{added}\right)} \times 100\% $$

### Cellulase activity analysis

Fermentation samples were collected and centrifuged at 14,000 g for 5 min at 4°C, and the supernatants were collected as the crude enzyme.

Hydroxyethyl cellulose (HEC), *p*-nitrophenyl-β-D-cellobiose (pNPC), *p*-nitrophenyl-β-D-glucoside (pNPG), glucose, and *p*-nitrophenol (pNP) (all purchased from Sigma, Beijing, China) were dissolved in sodium acetate buffer (0.1 M, pH 6.5) for the measurement of enzymatic activity, and both the crude enzyme and the buffer were equilibrated at 55°C. The filter paper activity was measured by using Whatman Grade 1 filter paper (GE Healthcare, Shanghai, China) as a substrate, as described by Dashtban *et al.* [20]; 3,5-dinitrosalicylic acid (DNS) was used for the determination of reducing sugars [27] with an ultraviolet spectrophotometer. Endoglucanase activity was measured with HEC as a substrate, and released glucose was measured using DNS, as described above. Exoglucanase and β-glucosidase activities were measured as described by Adelsberger *et al.* [28]. One unit of enzymatic activity was defined as the amount of glucose (mg) released by 1 mL of crude enzyme per minute. A Tecan Infinite 200 Pro multimode reader was used to detect the release of pNP from the substrate at 430 nm [20]. Standard curves for pNPC and pNPG were generated by using pNP as the standard. The standard curves for filter paper activity and endoglucanase were constructed using glucose and measured by the DNS method.

### Ethanol concentration analysis

Fermentation samples were collected, centrifuged at 14,000 g for 10 min, and filtered with a 0.45-μm filter. Ethanol concentrations were then measured using HPLC with an Aminex HPX-87H column (Bio-Rad, Hercules, CA, USA), as described by Du *et al.* [27]. The ethanol conversion ratio (%) is defined as the ratio of the ethanol weight produced compared to the theoretical yield based on the consumed carbon source, with theoretical yields defined as previously described [29].

### PCR-DGGE analysis

Total DNA was extracted from 5-mL fermentation samples using the E.Z.N.A. Soil DNA Kit (Omega Bio-Tek, Inc., Norcross, GA, USA) with a modified pretreatment as described by Li *et al.* [30] and stored at −20°C. PCR for DGGE analysis was performed using the 357 F-GC-clamp (5′-CGC CCG CCG CGC CCC GCG CCC GGC CCG CCG CCCCCG CCC CCC TAC GGG AGG CAG CAG-3′) as the forward primer and 518R (5′-ATT ACC GCG GCT GCT GG-3′) as the reverse primer with Hot-start Ex Taq (Takara Bio, China) DNA polymerase. The touchdown PCR program [31] was modified as follows. The PCR began with an initial melting step of 94°C for 3 min, followed by 20 cycles of 94°C for 30 s, annealing at 65°C for 30 s (decreasing 1°C per cycle), and extension at 72°C for 30 s. This step was followed by 10 cycles of 94°C for 30 s, 55°C for 30 s, and 72°C for 30 s, with a final elongation step of 10 min at 72°C. DGGE analysis of purified PCR products was performed on a DCode Universal Mutation Detection System (Bio-Rad, Hercules, CA) with a 6% (w/v) polyacrylamide gel in 0.5 × TAE using a 35 to 55% denaturing gradient (100% denaturant consisting of 40% v/v formamide and 7 M urea). The samples were loaded on gels and run at 60°C and 90 V for 12 hours. The gels were stained with ethidium bromide, and the images were captured using an AlphaImager 2200 system (Alpha Innotech, San Leandro, CA, USA).

The digitized DGGE images were analyzed with Quantity One image analysis software (Version 4.3.1, Bio-Rad Laboratories, Hercules, CA, USA), and the similarity of the gel patterns was analyzed using principal component analysis (PCA). DGGE bands were aligned and scored as present (score = 1) and absent (score = 0) as reported previously [32]. The score data was subsequently analyzed by SPSS software (SPSS v.20; SPSS Inc., Chicago, IL, USA) for PCA as described previously [33]. Each DGGE lane was analyzed as a variable in PCA, and a similarity matrix was calculated with the correlation coefficient matrix.

The target bands on the gels were excised and recovered using the QIAEX II Gel Extraction Kit (Qiagen, Manchester, UK), and then used as templates for PCR enrichment of fragments of each band with 357 F (5′-CCT ACG GGA GGC AGC AG-3′) as the forward primer and 518R (5′-ATT ACC GCG GCT GCT GG-3′) as the reverse primer [34] with Ex Taq. The PCR program began with an initial incubation at 95°C for 10 min, included 25 cycles of 94°C for 1 min, 50°C for 30 s, and 72°C for 1.5 min, and ended with a final extension step of 72°C for 5 min. The PCR products then were purified with an Agarose Gel DNA Purification Kit (Takara Biotechnology (Dalian) Co., Ltd.) and cloned using a T-Vector Kit (Takara Biotechnology (Dalian) Co., Ltd.) before being transformed into competent *Escherichia coli* DH5α cells [11]. Finally, clones were sequenced using an ABI 3730 sequencer according to the manufacturer’s instructions.

### Isolation of single strains

Isolation of single strains was performed as described previously [27]. Bacteria were operated in a DG250 anaerobic workstation (Don Whitley Scientific Limited, West Yorkshire, UK) and cultured on agar plates made by reinforced clostridial medium (RCM, CM0149, Oxoid; Thermo Fisher Biochemicals (Beijing) Ltd., Beijing, China) in anaerobic jars (10 Plate Polycarbonate Jar, A05077; Don Whitley Scientific Limited, UK) at 55°C for 3 days. Colonies with different phenotypes were subcultured three times. Isolated single strains were phylogenetically classified by 16S rRNA gene sequences with primer 27 F and 1492R [35]. The full-length 16S rRNA gene sequences for the identified strains mentioned in Table 2 are available from the National Center for Biotechnology Information (NCBI) as follows: *Pseudoxanthomonas taiwanensis* (KM036186), *Acetivibrio cellulolyticus* (KM036187), *Thermoanaerobacterium thermosaccharolyticum* (KM036188), *Clostridium stercorarium* (KM036189), *Clostridium thermosuccinogenes* (KM036190), *Clostridium thermopalmarium* (KM036191), and *Clostridium sporogenes* (KM036192)*.*

### Co-fermentation of consortia with *P. taiwanensis*

The consortia and single strains were separately cultured until they reached stationary phase, and the liquid cultures were then centrifuged and washed three times with PCS medium (lacking calcium carbonate and a carbon source). Seed cultures were initiated by mixing the consortium and single strains in different proportions, and the fermentation cultures with an initial inoculum of 10% in PCS medium were prepared in a gas atmosphere composed of 10% hydrogen, 10% carbon dioxide, and 80% nitrogen, and then cultured in incubators at 55°C. Samples were collected daily with syringes for HPLC and filter paper activity (FPA) analyses. Co-fermentations of consortia with other microbes were conducted similarly.

### β-glucosidase addition assay

β-glucosidase was purchased from Genencor and dialyzed with a selectively permeable polysulfone membrane (the molecular weight cut-off is 10,000 Da) in 200 volume ultrapure water for 30 min and repeated for a total of 5 times. The dialyzed β-glucosidase was added to HL-inoculated medium to reach final enzyme activities of 0.17, 0.34, 0.68, 1.36, 2.04, and 2.72 U/mL, with three samples at each concentration. Fermentation cultures were then incubated at 55°C, and samples were collected daily with syringes for ethanol titer measurement.

### *P. taiwanensis* performance analysis

*P. taiwanensis* was cultured in *Thermus* medium [17] with different carbon sources, including cellulose, xylan, cellobiose, D-fructose, sucrose, glucose, and xylose, in 55°C incubators. Culture supernatants were collected each day, and ethanol production and cellulase activities were analyzed as described above.

## Additional file

Additional file 1: Table S1. Consortium sampling regions.

## Abbreviations

CBP: consolidated bioprocessing
DGGE: denaturing gradient gel electrophoresis
FPA: filter paper activity
HPLC: high performance liquid chromatography
*P. taiwanensis*: *Pseudoxanthomonas taiwanensis*
PCA: principal component analysis
