# Supplementary material for: Cellulosic ethanol production by natural bacterial consortia is enhanced by Pseudoxanthomonas taiwanensis
Source: Biotechnol Biofuels. 2015 Jan 23;8:10. doi: 10.1186/s13068-014-0186-7 (PMC4308921; doi:10.1186/s13068-014-0186-7)
Supplement: Additional file 1: Table S1. — Consortium sampling regions. [file 13068_2014_186_MOESM1_ESM.doc]

**Additional file 1: Table S**1 Consortium sampling regions

| Consortium | Isolation source | Latitude and longitude | Region | Climate |
| --- | --- | --- | --- | --- |
| IS | Steppe soils | 108:58E 38:25N | Inner Mongolia | Temperate continental monsoon climate zone |
| HP | Paddy soils | 113:42E 34:44N | Henan | Semi-moist monsoon climate |
| HL | Forest humus | 109:41E 18:55N | Hainan | Tropical monsoon climate |
| SW | Wheat field | 116:31E 35:25N | Shandong | Temperate monsoon climate |
| JW | Wheat field | 116:37E 35:20N | Shandong | Temperate monsoon climate |
| BG | Goat dung | 116:09E 40:16N | Beijing | Temperate monsoon climate |
| WS | Sweet sorghum stalk | 108:04E 41:02N | Inner Mongolia | Temperate continental monsoon climate zone |
| HS | Forest humus | 109:46E 29:40N | Hunan | Continental humid subtropical monsoon climate |
| SP | Steppe soils | 113:08E 37:24N | Shanxi | Temperate monsoon climate |
| WP | Steppe soils | 108:04E 41:02N | Inner Mongolia | Temperate continental monsoon climate zone |
| BC | Cow dung | 116:09E 40:16N | Beijing | Temperate monsoon climate |
| NL | Leaf mold | 107:51E 22:20N | Guangxi | Subtropical monsoon climate |
| SL | Leaf mold | 110:39E 31:44N | Hubei | Subtropical humid monsoon climate |
| GL | Leaf mold | 110:18E 25:05N | Guangxi | Subtropical monsoon climate |
| JS | Sweet sorghum stalk | 113:08E 37:24N | Shanxi | Temperate monsoon climate |
| ZS | Sorghum stalk | 113:42E 34:44N | Henan | Semi-moist monsoon climate |
| SS | Sorghum stalk | 116:37E 35:20N | Shandong | Temperate monsoon climate |
